# Clinically inapparent mpox virus (MPXV) infections among clients of three anonymous Community Based Voluntary Counselling and Testing centres in Berlin, Germany, 2022–2023

**DOI:** 10.1186/s12879-024-09510-x

**Published:** 2024-06-20

**Authors:** Uwe Koppe, Klaus Jansen, Axel Jeremias Schmidt, Christoph Weber, Heike Schulze, Robert Kasimir Kulis-Horn, Carsten Tiemann, Ulrich Marcus

**Affiliations:** 1 Department of Infectious Disease Epidemiology, Robert Koch Institute, Berlin, Germany; 2 Deutsche Aidshilfe, Berlin, Germany; 3 Sigma Research, Department of Public Health, Environments and Society, London School of Hygiene and Tropical Medicine, London, UK; 4 Checkpoint BLN, Berlin, Germany; 5 MVZ Labor Krone, Bad Salzuflen, Germany

**Keywords:** Mpox, MPXV, Clinically inapparent infection

## Abstract

**Introduction:**

Since the mpox outbreak in 2022, it was unclear if and how often infections with mpox virus (MPXV) were clinically inapparent, i.e. not presenting to clinical care with mpox symptoms. Moreover, it was hypothesized that MPXV circulated in the affected communities before the outbreak was officially detected.

**Methods:**

We retrospectively tested rectal and urethral swabs, and pooled samples for presence of MPXV. Samples were obtained from routine STI testing of three anonymous Community Based Voluntary Counselling and Testing (CBVCT) centres in Berlin, in 2022 and 2023. Testing results were linked to anonymously provided behavioural data.

**Results:**

Overall, 9,053 samples from 6,600 client visits were included. Clinically inapparent MPXV infections were detectable in 1.1% of the samples. We did not find MPXV infections in the month before the first cases appeared in Berlin or between October 2022 and January 2023 when case numbers were low in Germany. However, during the outbreak period in 2022, we found clinically inapparent MPXV infections among 2.2% of the clients and during summer/autumn 2023 among 0.3%. The number of condomless anal/vaginal intercourse partners within the previous 6 months and PrEP use were identified as predictors of clinically inapparent MPXV infection.

**Conclusion:**

Clinically inapparent MPXV infections occurred during the mpox outbreak in Berlin in 2022 and post-outbreak in summer/autumn 2023. Unrecognized MPXV circulation in Berlin before the recognition of the outbreak in May 2022 appears unlikely. However, low-level sustained circulation of clinically inapparent MPXV infections need to be acknowledged in mpox prevention strategies.

## Introduction

During the multinational mpox outbreak, 3,800 persons with mpox diagnosis were reported through the statutory surveillance system in Germany between May 2022 and October 2023 [1, 2]. During that period, Berlin was the most affected federal state in Germany with 1,711 reported diagnoses (as of 11 January 2024) [3]. The first case in Berlin was recorded on 23 May 2022. Most cases were male and, where the information was available, indicated sex with other men as the probable mode of transmission [2]. Mpox cases frequently reported living with HIV or taking HIV pre-exposure prophylaxis (PrEP) [4, 5]. Moreover, previous or concurrent other STIs were also frequently reported [4, 5].

After the first mpox cases in Berlin were identified, it was hypothesised that undetected MPXV transmission might have already occurred in the weeks before [3]. Initial studies suggested that MPXV infection might in some cases remain asymptomatic or at least clinically inapparent, meaning that mpox is detectable without any symptoms or minimal symptoms that might be overlooked when not thoroughly examined. A study from Belgium found MPXV infection in three asymptomatic men after screening 224 samples from routine STI testing [6]. However, using an amended case definition and detailed inquiry of symptoms in a follow-up study, it remains unclear if truly asymptomatic mpox infections exist [7]. A French study found that 13 of 200 investigated anal swabs from people without mpox-specific symptoms tested positive for MPXV [8]. Moreover, modelling studies suggested that unrecognized mpox infections contribute to transmission dynamics [9, 10]. However, it was unclear, how often and under what circumstances clinically inapparent MPXV infections occur. It remains to be investigated to what extent persons with mild forms of mpox, who are not aware of their infection, might contribute to sustaining transmission, even after the number of symptomatic and notified mpox diagnoses have decreased.

In this study, we investigated if and how often MPXV could be detected in rectal, urethral and/or pooled samples from clients, who went for STI screening in three Community Based Voluntary Counselling and Testing centres (CBVCT) in Berlin and did not report mpox symptoms. We investigated whether clinically inapparent MPXV infections occurred before the first mpox cases were reported in May 2022, during the outbreak in 2022, and during two periods of low case numbers after the outbreak. In addition, we describe which of the routinely collected variables in the CBVCT centres were associated with the detection of clinically inapparent MPXV infections.

## Methods

### Study design and setting

We conducted a cross-sectional study to investigate the occurrence of clinically inapparent MPXV infections and which groups were affected.

In Berlin, anonymous STI testing is offered outside of the primary care system in three CBVCT centres that are friendly for men, who have sex with men (MSM). The attending clients are usually asymptomatic. Upon arrival in the CBVCT centres, the clients are asked to complete an anonymous questionnaire on sociodemographic, sexual behaviour and sexual health topics [11, 12]. In addition, rectal, urethral and pharyngeal swabs (separate or pooled), or urine samples are taken for gonorrhoea and chlamydia screening. Pooled samples are combined rectal, urethral, urine, and/or pharyngeal sample of one person.

Berlin is harbouring a large resident MSM population and is also an international travel destination for MSM visiting gay clubs, parties, and sex-on-premises venues [3, 13]. Berlin was the German city most affected by mpox in 2022 both in absolute and relative terms [1]. Thus, we considered it the most suitable German city to investigate clinically inapparent MPXV infections. Anonymous swab and pooled samples from the three CBVCT centres collected between 13 April 2022 – 31 January 2023 and 28 July – 31 October 2023, when mpox cases were notified again after months of no reported cases, were retrospectively analysed for the presence of MPXV DNA. We divided this timeframe into four periods: pre-outbreak, 13 April – 14 May 2022; outbreak, 15 May – 31 October 2022; post-outbreak 1, 1 November 2022 – 31 January 2023; and post-outbreak 2, 28 July 2023 – 31 October 2023. We analysed all available anal, urethral, and pooled samples during the pre-outbreak and outbreak periods. In the post-outbreak period 1, we analysed half of the samples (every other sample) between 1 November and 14 December 2022 since the number of notified cases in this time was very low and financial resources for sample testing were limited. Between 15 December 2022–31 January 2023, we returned to testing all available samples, because of local mpox clusters at the time. In the post-outbreak period 2, we analysed all available samples. MPXV testing results were matched with the anonymous questionnaire data for further analysis.

For comparison of the results from the screening study with the number of known mpox cases, we used statutory notification data from the Robert Koch-Institute from SurvStat 2.0 [1]. We restricted the data to the Federal State of Berlin and displayed the cases by month. Notification data are as of 04 January 2024.

### Participants and included samples

Residual samples from anal, urethral, and pooled samples from clients of three Berlin CBVCT centres in Berlin were included in the analysis. For some clients, multiple samples were available. Samples from women and vaginal samples were not tested since their risk of mpox infection during that period was low. Clients without a residual sample were also excluded from the analysis.

### Outcome and laboratory procedures

The main outcome of the study was the presence of clinically inapparent MPXV infection as determined by presence of MPXV DNA in residual samples from previous STI screening. The samples (rectal swabs, urethral swabs, pharyngeal swabs, pooled samples) were stored in Aptima transport buffer (Hologic, USA) ensuring DNA stability between sampling date and retrospective MPXV testing. Magnetic beads were used for viral DNA extraction, followed by MPXV-specific real-time PCR detection using the Novaplex™ MPXV Assay (Seegene, Korea). An endogenous control (human DNA) was used to confirm the validity of negative MPXV PCR results.

In case of a positive result, CBVCT centres were asked to confirm that no symptoms had been documented for that client at the time when the sample was taken. Clients with at least one positive sample and without recorded symptoms were considered to have clinically inapparent MPXV infection.

### Variables and statistical analysis

For analyses over time, we used the date when the sample was taken. If that was not available, we used the date when the sample arrived in the laboratory.

For the selection of participants for analysis of the questionnaire data, we used information reported by the clients on their gender identity (male, female, transgender male, transgender female, non-binary, another gender identity) as well as sexual orientation (gay, lesbian, heterosexual, queer, bisexual, another orientation).

We stratified the results by CBVCT centre as well as by information provided in the questionnaire. This included age in categories of < 29, 30–39, 40–49, and 50 + years, as well as gender in categories of cisgender male, transgender male, transgender female, non-binary and another gender identity. The number of sexual partners within the previous six months was reported in categories of 0, 1–4, and 5+. Moreover, we report HIV-PrEP use, concurrent gonorrhoea or chlamydia infection in categories of yes or no. Syphilis was analysed in categories of negative, residual antibodies and active syphilis. Wherever information was missing, it was coded as unknown.

In order to reduce misclassification of potentially symptomatic mpox clients as clinically inapparent, we asked the testing sites to re-check their records if any symptoms had been documented for clients with samples testing positive for MPXV DNA. Four clients with MPXV detected in the stored samples were excluded due to mpox symptoms. One client’s sample tested negative, but was recorded to be treated in one CBVCT centre due to mpox symptoms and tested positive for mpox there.

Variables were analysed descriptively using percentages for categorical variables and medians with interquartile ranges (IQR) for continuous variables. The association of the covariables with the outcome of clinically inapparent MPXV infection was investigated using χ2-tests for comparison of categorical variables and Wilcoxon rank-sum tests for comparison of medians of continuous variables. Comparisons of percentages between categories and χ2-tests were performed excluding missing or unknown data. We identified meaningful predictors for clinically inapparent MPXV infection using the bootstrap stepwise selection method as previously described [14]. In brief, we performed 500 bootstrap replications, in which we selected potential predictors in a multivariable logistic regression model using the backward modelling approach with a p-value cut-off of 0.1. We then report the absolute and relative inclusion frequencies of the potential predictors into the multivariable models. Variables included in > 80% of the multivariable models were considered as predictive. Variables investigated as potential predictors include age, gender, number of sexual partners, number of sexual partners with condomless anal/vaginal intercourse, PrEP use, as well as active syphilis, gonorrhoea or chlamydia infection. Missing data were reported for all variables and excluded in statistical comparisons. All analyses were carried out using Stata 17.0 (Stata Statistical Software: Release 17, USA).

## Results

### Identification of clinically inapparent MPXV infections

Overall, we tested 9,082 samples from the observational period. After exclusion of 22 samples with inconclusive results, 5 samples of clients with recorded symptoms and 2 duplicate samples, we included 9,053 samples of 6,600 clients in our analysis. Thus, we analysed 686 samples of 487 clients during the pre-outbreak period (13 April – 14 May 2022), 4,380 samples of 3,112 clients during the outbreak 2022 period (15 May – 31 October 2022), 1,678 samples of 1,344 clients during the post-outbreak period 1 (01 November 2022 – 31 January 2023), and 2,309 samples of 1,657 clients during the post-outbreak period 2 (28 July – 31 October 2023) (Table 1).

**Table 1:** Anonymous rectal and/or urethral samples tested for mpox and testing results

Period	Month	Number of tested samples with interpretable result (*n*)	Number of tested clients (*n*)	Number of clients with positive MPXV-result, *n* (%)
Pre-outbreak	April 2022	332	242	0 (0.0%)
	May 2022^1^	354	245	0 (0.0%)
Outbreak 2022	May 2022^2^	437	316	11 (3.5%)
	June 2022	817	572	33 (5.8%)
	July 2022	728	492	19 (3.9%)
	August 2022	813	571	5 (0.9%)
	September 2022	806	590	2 (0.3%)
	October 2022	779	571	0 (0.0%)
Post-outbreak 1	November 2022	382	346	0 (0.0%)
	December 2022	488	402	0 (0.0%)
	January 2023	808	596	0 (0.0%)
Post-outbreak 2	July/August 2023^3^	859	613	2 (0.3%)
	September 2023	686	473	1 (0.2%)
	October 2023	764	571	2 (0.4%)

^1^ 1 – 14 May 2022; ^2^ 15 – 31 May 2022; ^3^ 28 July – 31 August 2023

Overall, we detected 75/6,600 clients (1.1%) with clinically inapparent mpox (Table 1). No cases of clinically inapparent MPXV infections were detected during the pre-outbreak period. During the outbreak 2022 period, clinically inapparent MPXV infections were found between 17 May 2022 and 27 September 2022, peaking at 5.8% of the investigated samples in June. The proportion of clients with clinically inapparent MPXV infections during the outbreak 2022 period was 2.2% (70/3,112) of the investigated samples. During the post-outbreak 1 period between November 2022 and January 2023, no clients with clinically inapparent MPXV infections were found. However, during the post-outbreak period 2 from 28 July to 31 October 2023, samples of 5/1,657 (0.3%) clients tested positive for MPXV.

**Fig. 1:**
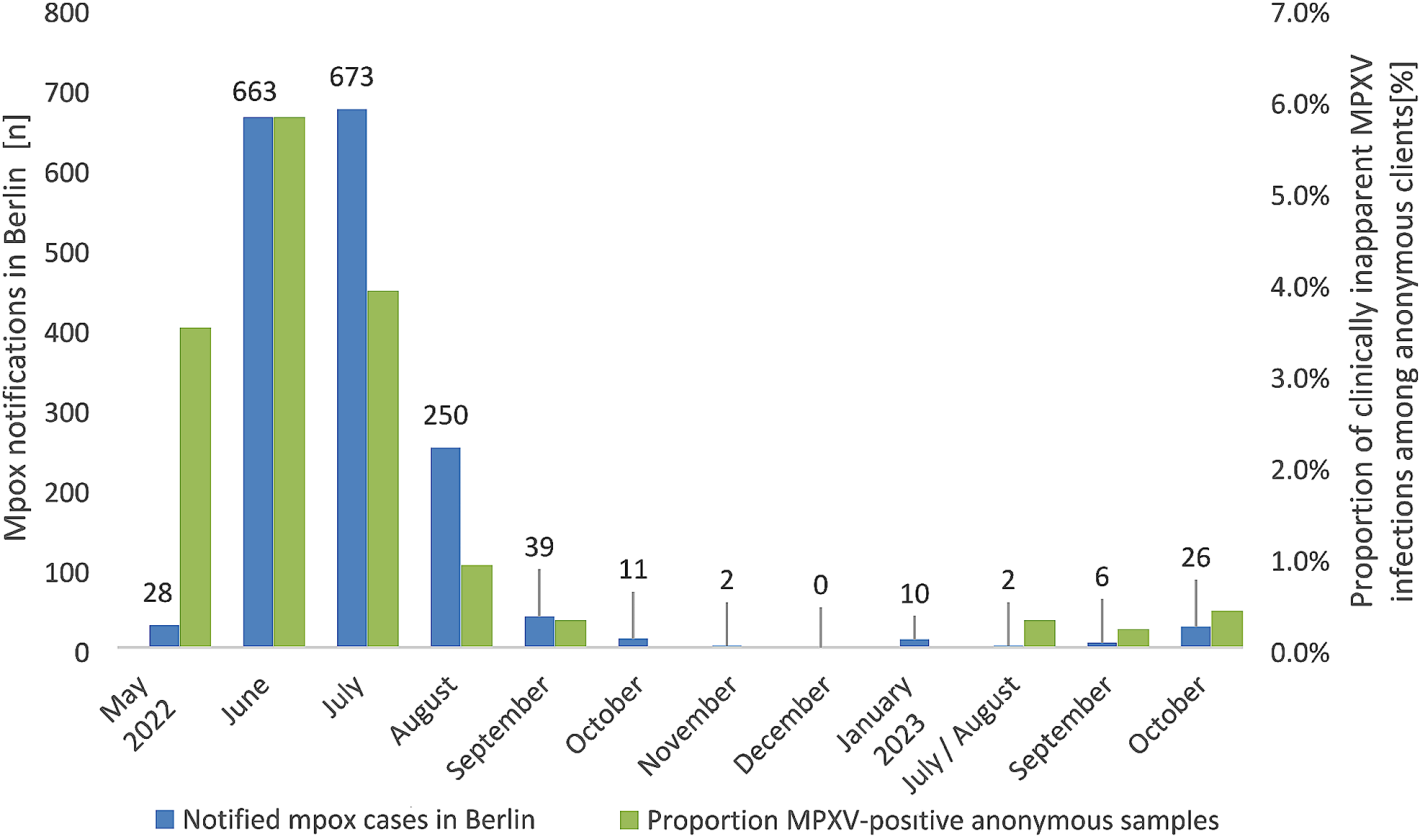
Notified mpox cases in Berlin during the outbreak and post-outbreak periods and proportion of CBVCT clients with clinically inapparent MPXV infections based on retrospective testing of anal/urethral swab and urine samples. Numbers above bars represent Mpox notifications in Berlin. Values for proportions of clinically inapparent MPXV infections are shown in Table 1. The category “May 2022” includes data between 15 May – 31 May 2022, the category “July/August 2023” between 28 July − 31 August 2023

The period with the highest proportion of clinically inapparent MPXV infections from mid-May 2022 to July 2022 was also the period with the highest numbers of mpox notifications in the statutory surveillance system (Fig. 1). From October 2022 to January 2023, we did not detect any clinically inapparent mpox cases while 23 mpox cases were notified in the surveillance system in that time period. This dynamic changed during the post-outbreak period 2, when 34 mpox cases were notified in Berlin, while 5/1,657 (0.3%) of clients from the anonymous sampling were found with clinically inapparent MPXV infections.

### Analysis of potential predictors for clinically inapparent MPXV infections

**Fig. 2:**
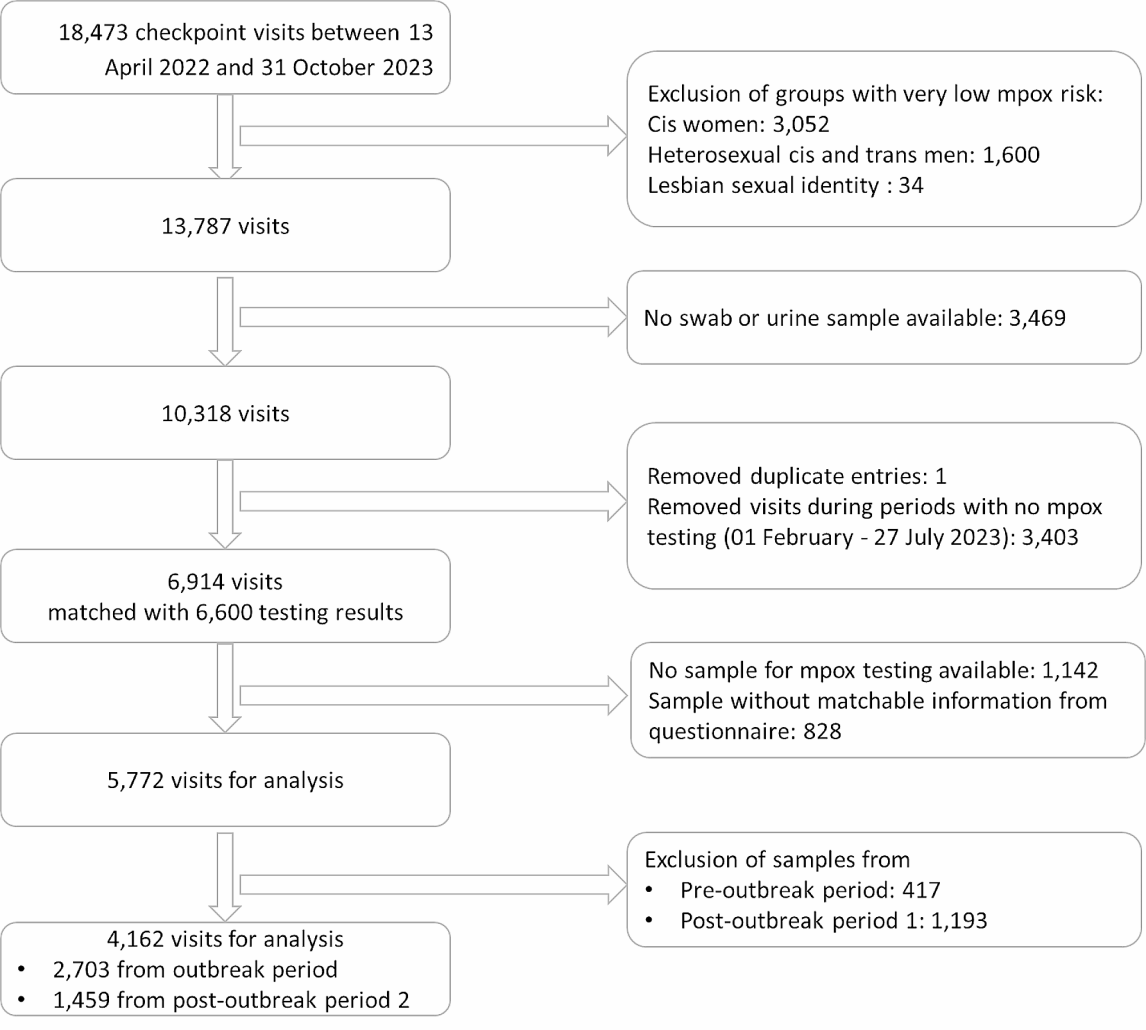
Flowchart of in- and excluded CBVCT clients / specimens

To obtain more information on clients with clinically inapparent MPXV infections, we analysed the data provided by the clients through an online questionnaire while attending the testing site. We only analysed data from the outbreak period and the post-outbreak period 2 since these were the only periods with clinically inapparent MPXV infections in our samples. Out of data from 18,473 visits, we excluded data from clients, who were not part of the groups affected by mpox (cisgender women, lesbian women, heterosexual cis and trans men) as well as those without a sample for analysis of mpox infection (Fig. 2). After further exclusion of visits without samples, duplicate entries, visits or samples that could not be matched and observational periods not investigated in this section, we remained with 2,703 visits with corresponding samples tested for MPXV between 15 May 2022 – 31 October 2022 and 1,459 visits between 28 July – 31 October 2023.

**Table 2:** Characteristics and mpox testing results among clients of three CBVCT centres in Berlin, Germany

	Outbreak period: 15 May – 31 Oct 2022			Post-outbreak period 2: 28 July – 31 Oct 2023		
	Clients, *n*(%)	Clients testing negative for MPXV DNA, *n*(%)	Clients testing positive for MPXV DNA, *n*(%)	Clients, *n*(%)	Clients testing negative for MPXV DNA, *n*(%)	Clients testing positive for MPXV DNA, *n*(%)
Total	2,703 (100.0%)	2,639 (100.0%)	64 (100.0%)	1,459 (100.0%)	1,455 (100.0%)	4 (100.0%)
Testing site 1	60 (2.2%)	59 (2.2%)	1 (1.6%)	29 (2.0%)	29 (2.0%)	0 (0.0%)
Testing site 2	1,122 (41.5%)	1,102 (41.8%)	20 (31.3%)	523 (35.8%)	522 (35.9%)	1 (25.0%)
Testing site 3	1,521 (56.3%)	1,478 (56.0%)	43 (67.2%)	907 (62.2%)	904 (62.1%)	3 (75.0%)
Age						
Median (IQR)	33 (28–40)	33 (28–39)	35 (31–43)	33 (28–40)	33 (28–40)	31.5 (30–36)
<29 years	851 (31.5%)	840 (31.8%)	11 (17.2%)	437 (30.0%)	436 (30.0%)	1 (25.0%)
30–39 years	1,160 (42.9%)	1,128 (42.7%)	32 (50.0%)	643 (44.1%)	641 (44.1%)	2 (50.0%)
40–49 years	406 (15.0%)	394 (14.9%)	12 (18.8%)	206 (14.1%)	205 (14.1%)	1 (25.0%)
50 + years	269 (10.0%)	260 (9.9%)	9 (14.1%)	162 (11.1%)	162 (11.1%)	0 (0.0%)
Unknown	17 (0.6%)	17 (0.6%)	0 (0.0%)	11 (0.8%)	11 (0.8%)	0 (0.0%)
Gender identity						
Cisgender male	2,424 (89.7%)	2,363 (89.5%)	61 (95.3%)	1,281 (87.8%)	1,277 (87.8%)	4 (100.0%)
Transgender male	16 (0.6%)	16 (0.6%)	0 (0.0%)	19 (1.3%)	19 (1.3%)	0 (0.0%)
Transgender female	32 (1.2%)	32 (1.2%)	0 (0.0%)	9 (0.6%)	9 (0.6%)	0 (0.0%)
Non-binary	192 (7.1%)	191 (7.2%)	1 (1.6%)	130 (8.9%)	130 (8.9%)	0 (0.0%)
Another gender identity	18 (0.7%)	17 (0.6%)	1 (1.6%)	11 (0.8%)	11 (0.8%)	0 (0.0%)
Unknown gender identity	21 (0.8%)	20 (0.8%)	1 (1.6%)	9 (0.6%)	9 (0.6%)	0 (0.0%)
Residing in Germany						
No	269 (10.0%)	260 (9.9%)	9 (14.1%)	177 (12.1%)	175 (12.0%)	2 (50.0%)
Yes	1,316 (48.7%)	1,279 (48.5%)	37 (57.8%)	702 (48.1%)	701 (48.2%)	1 (25.0%)
Unknown	1,118 (41.4%)	1,100 (41.7%)	18 (28.1%)	580 (39.8%)	579 (39.8%)	1 (25.0%)
Number of sexual partners within the previous 6 months						
0	13 (0.5%)	13 (0.5%)	0 (0.0%)	3 (0.2%)	3 (0.2%)	0 (0.0%)
1–4	875 (32.4%)	868 (32.9%)	7 (10.9%)	480 (32.9%)	479 (32.9%)	1 (25.0%)
5+	1,783 (66.0%)	1,727 (65.4%)	56 (87.5%)	955 (65.5%)	952 (65.4%)	3 (75.0%)
Unknown	32 (1.2%)	31 (1.2%)	1 (1.6%)	21 (1.4%)	21 (1.4%)	0 (0.0%)
Number of sexual partners with condomless anal or vaginal intercourse (CAVI) within the previous 6 months						
0	333 (12.3%)	332 (12.6%)	1 (1.6%)	198 (13.6%)	198 (13.6%)	0 (0.0%)
1–4	1,200 (44.4%)	1,189 (45.1%)	11 (17.2%)	633 (43.4%)	632 (43.4%)	1 (25.0%)
5+	624 (23.1%)	585 (22.2%)	39 (60.9%)	379 (26.0%)	377 (25.9%)	2 (50.0%)
Unknown	546 (20.2%)	533 (20.2%)	13 (20.3%)	249 (17.1%)	248 (17.0%)	1 (25.0%)
PrEP use						
No	1,778 (65.8%)	1,760 (66.7%)	18 (28.1%)	921 (63.1%)	921 (63.3%)	0 (0.0%)
Yes	810 (30.0%)	769 (29.1%)	41 (64.1%)	468 (32.1%)	465 (32.0%)	3 (75.0%)
Unknown	115 (4.3%)	110 (4.2%)	5 (7.8%)	70 (4.8%)	69 (4.7%)	1 (25.0%)
Syphilis						
No	1,773 (65.6%)	1,757 (66.6%)	16 (25.0%)	943 (64.6%)	942 (64.7%)	1 (25.0%)
Yes, residual antibodies	233 (8.6%)	215 (8.1%)	18 (28.1%)	157 (10.8%)	157 (10.8%)	0 (0.0%)
Yes, concurrent syphilis	55 (2.0%)	52 (2.0%)	3 (4.7%)	21 (1.4%)	20 (1.4%)	1 (25.0%)
Unknown	642 (23.8%)	615 (23.3%)	27 (42.2%)	338 (23.2%)	336 (23.1%)	2 (50.0%)
Concurrent Gonorrhea						
No	2,439 (90.2%)	2,388 (90.5%)	51 (79.7%)	1,269 (87.0%)	1,266 (87.0%)	3 (75.0%)
Yes	264 (9.8%)	251 (9.5%)	13 (20.3%)	190 (13.0%)	189 (13.0%)	1 (25.0%)
Concurrent Chlamydia						
No	2,473 (91.5%)	2,422 (91.8%)	51 (79.7%)	1,337 (91.6%)	1,334 (91.7%)	3 (75.0%)
Yes	230 (8.5%)	217 (8.2%)	13 (20.3%)	121 (8.3%)	120 (8.2%)	1 (25.0%)
Unknown	-	-	-	1 (0.1%)	1 (0.1%)	0 (0.0%)

During the outbreak 2022 period, 2,703 client visits with questionnaire data were analysed, including 64 clinically inapparent MPXV infections (Table 2). Most clients attended predominantly two testing sites. The median age of all clients was 33 years (IQR 28–40). Clients with clinically inapparent MPXV infection were slightly older (35 years, IQR: 31–43) than those without (33 years, IQR: 28–39, *p* = 0.008). Almost all clinically inapparent MPXV infections occurred among cisgender men (Table 2).

Where information was available, clients with clinically inapparent MPXV infections more often reported PrEP use than clients without (69.5% vs. 30.4%, *p* < 0.001). Moreover, clients with clinically inapparent MPXV infections were more often reporting 5 or more sexual partners within the previous 6 months than clients without (88.9% vs. 66.2%, *p* < 0.001). The difference was even more pronounced when comparing the proportion of 5 or more sexual partners with condomless anal or vaginal intercourse (CAVI) within the previous 6 months between clients with and without clinically inapparent MPXV infection (76.5% vs. 27.8%, *p* < 0.001). In addition, clients with clinically inapparent MPXV infections more often had residual syphilis antibodies (48.6% vs. 10.6% ) or concurrent active syphilis (8.1% vs. 2.6%) (*p* < 0.001 for overall comparison), concurrent gonorrhoea (20.3% vs. 9.5%, *p* = 0.004), and concurrent chlamydia infection (20.3% vs. 8.2%, *p* = 0.001). We identified 487/2,703 client visits with a diagnosis of active syphilis, gonorrhoea, and/or chlamydia. Being diagnosed with any of these STI was more common among clients with clinically inapparent MPXV infection than those without (37.5% vs. 17.5%, *p* < 0.001).

**Table 3:** Absolute and relative inclusion frequencies of potential predictors for clinically inapparent MPXV infection during the outbreak period after 500 replications (*n* = 1.605)

	Outbreak period 2022	
Age	44	8.8%
Gender	0	0%
Number of sexual partners in the previous 6 months	45	9.0%
Number of sexual partners with condomless anal or vaginal intercourse in the previous 6 months	466	93.2%
PrEP use	400	80.0%
Concurrent syphilis	9	1.8%
Concurrent gonorrhoea	102	22.2%
Concurrent chlamydia	40	8.0%

To identify predictors for clinically inapparent MPXV infections during the outbreak period, we determined absolute and relative inclusion frequencies of potential predictors in multivariable models using bootstrapping as described. Potential predictors were age, gender, the number of sexual partners overall and with condomless anal/vaginal intercourse, PrEP use, active syphilis as well as concurrent gonorrhoea and chlamydia infections. The number of sexual partners with condomless anal/vaginal intercourse within the previous 6 months was included as a predictor in 93.2% of the models and PrEP use was identified as a predictor in 80.0% of the models. Both variables were thus identified as suitable predictors for clinically inapparent MPXV infection (Table 3). The inclusion frequencies of the other variables were all below 23% (Table 3).

The client characteristics during the post-outbreak period were comparable to those in the outbreak period (Table 2). Overall, four clinically inapparent MPXV infections during 1,459 client visits were identified. Due to the small number of cases, we cannot make statistical comparisons between clients with and without clinically inapparent MPXV infection in this time period.

## Discussion

In this study on retrospective MPXV-screening of anonymous samples from CBVCT clients in Berlin, Germany, we could not detect MPXV circulation in the month before the notification of the first mpox case in Berlin in May 2022 or during the post-outbreak period 1. However, during the outbreak 2022 period, we found clinically inapparent MPXV infections in samples of 2.2% of the investigated population and during the post-outbreak period 2 in 0.3% of the clients. During the outbreak period, clients with 5 or more sexual partners with CAVI within the previous 6 months, those using PrEP, and those with concurrent STIs were more often affected by clinically inapparent MPXV infection. Suitable predictors for clinically inapparent MPXV infection were the number of partners with condomless anal/vaginal intercourse and PrEP use.

Even though epidemiological data from the early phase of the large mpox outbreak in Berlin showed that many cases were travel-associated, it was unclear if there might have been unrecognized circulation of MPXV before the outbreak was detected [3]. In our study, we could not find evidence for clinically inapparent MPXV infection among any of the 486 clients who visited one of the three sites CBVCT centres in Berlin for STI screening during the month before the first cases in Berlin were reported. This finding is similar to the results from a Dutch study, that did not find evidence of MPXV infection circulation in Amsterdam or Rotterdam prior to the mpox outbreak [15]. While it is entirely possible that some mpox cases might have been missed in the early stage of the mpox outbreak, it appears plausible that most cases in Berlin in May 2022 were travel-associated, followed by a change to autochthonous transmission [3].

During the outbreak period, we found clinically inapparent MPXV infection among 2.2% of CBVCT clients. In a study in Paris, France, 13 out of 200 MSM (6.5%) without mpox symptoms tested positive for MPXV between 5 June and 11 July after testing anal swabs of MSM [8]. Two of these reported developing symptoms at a later stage. In our study, we also found a prevalence of clinically inapparent MPXV infections of similar magnitude in rectal and pooled samples that were collected in June 2022. A Japanese study tested 4/1,346 asymptomatic participants (0.3%) positive for MPXV, three of which remained asymptomatic for a month while one person developed symptoms after the test [16]. In a study from Antwerp, Belgium, 3 out of 224 MSM (1.3%) tested positive for MPXV infection without reporting mpox-specific symptoms and one person tested positive, who had symptoms that were misdiagnosed as herpes simplex [6]. A follow-up study found 14 undiagnosed mpox cases among 327 screened MSM (4.3%) [7]. Even though most of these undiagnosed cases might have lacked mpox-specific skin lesions, all undiagnosed cases had some symptoms, e.g. proctitis or lymphadenopathy, upon further inquiry or developed mpox-specific symptoms in the days after the positive test. In our study, we ensured that we only included clients that did not report symptoms at the time of sampling. However, due to the anonymous study design we did not have the possibility to retrospectively ask for symptoms or assess, if clients might have developed symptoms later on.

When analysing the occurrence of clinically inapparent MPXV infections in 2022, we found evidence for such infections during the outbreak period in times of higher numbers of notified mpox cases. Thus, clinically inapparent MPXV infections mostly appeared to be occurring alongside symptomatic mpox infections in our study setting. Considering data from other screening studies it is possible that people with clinically inapparent MPXV infections might develop symptoms at later time points. During the outbreak period, clinically inapparent MPXV infections occurred more often among clients with high numbers of condomless sexual partners, PrEP users, as well as clients with a history of syphilis and/or concurrent STIs. Moreover, we identified the number of partners with condomless anal/vaginal intercourse within the previous 6 months and PrEP use as suitable predictors. This is similar to the findings in the Belgian and French studies mentioned above [6–8]. In these groups symptomatic MPXV infections were also often observed [4, 5]. Thus, CAVI might be a probable mode of transmission of sexually transmitted MPXV. Consequently, condom use may play a role in reducing sexual transmission in people at increased risk of MPXV infections, especially in situations of exclusive genito-anal/vaginal contact.

During the post-outbreak period 2, we identified a low number of clinically inapparent MPXV infections. While the characteristics of the CBVCT clients during that period appear comparable to the outbreak period, the reported number of mpox cases in the statutory surveillance system was much lower. We can only speculate on the reasons for the occurrence of clinically inapparent MPXV infections during this period with low numbers of reported mpox cases. Due to the roll-out of mpox vaccinations, some vaccinated people might experience only mild forms of mpox infection [17]. If that would increase the likelihood of clinically inapparent MPXV infections is currently not known, but could be a possible explanation for our findings, if this were the case. Since data on mpox vaccination is not available, we cannot further investigate this hypothesis in this study setting. Since July 2023 low numbers of mpox cases are reported in Germany, which might indicate that ongoing low-level transmissions occurs. As most of the notified cases were reported without epidemiological links, it could be assumed that there might be a distinct proportion of inapparent MPXV infections triggering this constant low-level transmission.

Our findings contribute important information for public health and prevention strategies. We found that some MPXV infections can be clinically inapparent at least at some stage of infection, especially without a specific inspection e.g. of the rectum, the pharynx or any other body part that is not easy to observe from a patient’s perspective. These results are in line with previously published studies [9, 10]. Persons with clinically inapparent infections might contribute to the transmission dynamics, which needs to be considered in terms of prevention campaigns and recommendations. This contribution might be substantial since it is conceivable that persons with clinically inapparent MPXV infection might not limit their sexual behaviour and thus could be more likely to pass on the infection.

A strength of our study is the large sample size and the long time period that we were able to investigate. Moreover, we could link the testing results to questionnaire data so that we could provide further context on the clients affected by clinically inapparent MPXV infection.

We also need to consider some limitations. It is possible that some clients with mild and unnoticed symptoms might have been misclassified as “not symptomatic” and included in our study. However, this does not impact the conclusions drawn from our analyses since this would reflect a real-world situation of clinically inapparent MPXV infections where people were unaware of mild symptoms and would not report them to a physician or testing centre. Another limitation is that due to anonymous study data we have no clinical follow-up information so that we cannot know if some clients might have developed noticeable symptoms at a later stage. Thus, our study data do not allow us to distinguish between pre-symptomatic and asymptomatic MPXV infections. Since CBVCT clients remain anonymous, it is possible that some clients might have been included multiple times over the observational period. However, this does not impact our conclusions on the circulation of MPXV in the community over time, or on clinically inapparent MPXV infections.

In addition, the study population consists of CBVCT clients, who might not be completely representative of the total population at risk for mpox [12]. Thus, the results might not be applicable to other vulnerable groups for mpox (e.g. household contacts of cases).

Lastly, we only tested anal, urethral or pooled samples. We did not include separate testing of pharyngeal samples over the whole observational period. After testing pharyngeal samples from June 2022, when case numbers where highest in Berlin, the inclusion of pharyngeal samples would have increased the number of identified cases by 3 from 33 to 36. Due to limited resources and an only marginally increased amount of identified inapparent MPXV infections, we decided to forego further testing of pharyngeal samples. Thus, isolated MPXV infections in the pharynx may have been overlooked by our study.

## Conclusion

Clinically inapparent mpox infections could be detected during the mpox outbreak in Berlin in 2022 and during the post-outbreak period 2 in summer/autumn 2023. Thus, they need to be considered in public health strategies for testing and prevention. Moreover, the likelihood of clinically inapparent MPXV infections in the context of mpox vaccination needs to be further elucidated.

## Data Availability

The data analysed in our study are available through the corresponding author on reasonable request.

## Abbreviations

CAVI: Condomless anal or vaginal intercourse
CBVCT: Community Based Voluntary Counselling and Testing Centres
IQR: Interquartile range
MSM: Men, who have sex with men
MPXV: Mpox virus
PrEP: HIV pre-exposure prophylaxis
STI: Sexually transmitted infection
